# Usability and acceptability of self-testing for hepatitis C virus infection among the general population in the Nile Delta region of Egypt

**DOI:** 10.1186/s12889-021-11169-x

**Published:** 2021-06-22

**Authors:** Elena Ivanova Reipold, Ahmed Farahat, Amira Elbeeh, Reham Soliman, Elkin Bermudez Aza, Muhammad S. Jamil, Cheryl Case Johnson, Gamal Shiha, Philippa Easterbrook

**Affiliations:** 1 FIND, Geneva, Switzerland; 2 Egyptian Liver Research Institute and Hospital (ELRIAH), Mansoura, Egypt; 3 Association of Liver Patient Care (ALPC), Mansoura, Egypt; 4 Tropical Medicine Department, Port Said University, Port Said, Egypt; 5 Department of Global HIV, Hepatitis and STI Programmes, World Health Organization, Geneva, Switzerland; 6 Internal Medicine Department, Mansoura University, Mansoura, Egypt

**Keywords:** Hepatitis C virus, Rapid diagnostic tests, Self-test, HCV, Usability, Acceptability

## Abstract

**Background:**

Self-testing for hepatitis C virus antibodies (HCVST) may be an additional strategy to expand access to hepatitis C virus (HCV) testing and support elimination efforts. We conducted a study to assess the usability and acceptability of HCVST among the general population in a semi-rural, high-HCV prevalence region in Egypt.

**Methods:**

An observational study was conducted in two hospitals in the Nile Delta region. A trained provider gave an in-person demonstration on how to use the oral fluid HCVST followed by observation of the participant performing the test. Usability was assessed by observing errors made and difficulties faced by participants. Acceptability of HCV self-testing was assessed using an interviewer-administered semi-structured questionnaire.

**Results:**

Of 116 participants enrolled, 17 (14.6%) had received no formal education. The majority (72%) of participants completed all testing steps without any assistance and interpreted the test results correctly. Agreement between participant-reported HCVST results and interpretation by a trained user was 86%, with a Cohen’s kappa of 0.6. Agreement between participant-reported HCVST results and provider-administered oral fluid HCV rapid test results was 97.2%, with a Cohen’s kappa of 0.75. The majority of participants rated the HCVST process as easy (53%) or very easy (44%), and 96% indicated they would be willing to use HCVST again and recommend it to their family and friends.

**Conclusion:**

Our study demonstrates the high usability and acceptability of oral fluid HCVST in a general population. Further studies are needed to establish the optimal positioning of self-testing alongside facility-based testing to expand access to HCV diagnosis in both general and high-risk populations.

**Supplementary Information:**

The online version contains supplementary material available at 10.1186/s12889-021-11169-x.

## Introduction

Hepatitis C virus (HCV) infection is a major cause of chronic liver disease worldwide. An estimated 71 million individuals are chronically infected with HCV, and there is a disproportionately high burden of this disease in low- and middle-income countries (LMICs) [1]. The global response to HCV has been transformed with the introduction of curative, short-course, pan-genotypic direct-acting antiviral (DAA) therapy. This has led to the adoption of a “treat all” approach for HCV-infected persons, regardless of disease stage, and available at low cost in most LMICs. In 2016, the World Health Organization (WHO) launched the Global Health Sector Strategy on Hepatitis 2016–2021, with the ambitious goal to eliminate HCV as a public health threat by 2030 [2]. There has been considerable scale-up of testing and treatment in several champion countries, in particular Egypt [3]; however, globally, less than 20% of all persons with HCV infection have been tested and less than one-quarter of diagnosed patients have been treated [1]. This gap in diagnosis and treatment is even higher in many LMICs that have a high burden of HCV. This is particularly true in rural or hard to reach settings and among some high-risk groups, such as people who inject drugs (PWID) and men who have sex with men (MSM).

WHO recommends focused screening for HCV infection in the most affected populations in all settings and routine testing of all adults, adolescents and children in settings with ≥2% HCV antibody prevalence in the general population [4, 5]. In addition, WHO recommends a single rapid diagnostic test (RDT) followed by prompt HCV RNA viral load test to confirm viremia and staging of liver disease prior to initiating treatment [4, 5]. Lack of access to HCV testing services and confirmatory viral load testing remain significant barriers to expanding treatment efforts. To expand access to HCV testing and treatment will require greater decentralization of testing and treatment services to primary care and harm reduction sites [6], in addition to the adoption of innovative and convenient testing approaches, including self-testing [7].

Self-testing, where people collect their own specimen, perform a simple rapid test, and interpret the result, has been recommended by WHO since 2016 [8], as an accurate, safe, and acceptable approach to reach people with human immunodeficiency virus (HIV) who may not otherwise access testing, including high-risk populations [9–12]. Most untrained lay users can perform HIVST as effectively as trained providers, and adverse events are rare [13, 14]. HIV self-testing (HIVST) national policy uptake has grown rapidly - 88 countries had HIVST friendly polices as of July 2020, and 41 of them were routinely implementing HIVST [15]. In 2019, WHO updated its guidance on HIVST, highlighting service delivery models and support tools to assist further implementation and scale-up [16].

Self-testing for HCV antibody (HCVST) may be an additional strategy to support elimination as it could increase the coverage of HCV testing reaching individuals missed by conventional facility or community-based testing modalities. Self-testing offers privacy and convenience. However, HCVST also raises important concerns particularly with challenges in conduct and interpretation of test results due to user errors, and in linking individuals with positive self-test results to further confirmatory testing and care. Current experience with HCVST remains limited to small pilot or research studies. These include a qualitative study of self-sampling among 22 PWID in London [17] and two studies, conducted in the United States and China, on the accuracy of oral fluid-based HCV antibody tests adapted for self-testing [18, 19]. These studies showed high agreement between results obtained by untrained users and healthcare provider-delivered testing [18, 19]. The use of HCVST has not yet been recommended by WHO, and as yet there have been no products approved by a stringent regulatory authority in any country or prequalified by WHO. However, several professional-use HCV RDTs already prequalified by WHO could potentially be adapted by the manufacturers for self-testing use. Further studies on the usability and acceptability of HCVST in different population groups and settings are needed to inform global guidelines and policy development.

FIND (Foundation for Innovative New Diagnostics), in collaboration with WHO, has recently undertaken an initial series of pilot studies to examine the usability and acceptability of HCVST in a range of different settings across five countries – Egypt (general population in a high burden country), China (MSM), Vietnam (PWID and MSM), Georgia (PWID and MSM), and Kenya (PWID). These sites and populations were selected for these small pilot studies (less than 150 individuals) based on existing collaborative partnerships on HCV testing and treatment, and not on the intended prioritisation of these countries for implementation and promotion of HCVST. We report here an assessment of the usability and acceptability of HCVST among the general population in a semi-rural setting in Egypt. Egypt has among the highest prevalence and burden of HCV infection worldwide, with a generalized epidemic, largely as a result of poor injection safety and other unsafe medical practices [20]. In 2015, the estimated national prevalence of chronic viremic HCV infection was 7% in those aged 15 to 59 years [21]. The national government and the Ministry of Health and Population (MOHP) [22] established an early, effective viral hepatitis response, with the goal of eliminating HCV infection from Egypt [23]. In October 2018, the Egypt government initiated mass screening of the population to improve case-finding, under the Presidential Initiative “100 Million Healthy Lives”. As of December 2019, more than 50 million Egyptians had been tested [23], almost two-thirds of the national population, and a total of 3 million people have been treated with DAAs since 2014.

## Methods

### Study design and setting

We undertook a cross-sectional, observational study in two hospitals (Association of Liver Patient Care Hospital (ALPC) and Shirbin Hospital) in the Nile Delta region of the Mansoura region of Egypt. Both hospitals are associated with the Egyptian Liver Research Institute and Hospital (ELRIAH), a non-governmental organization focused on the development and dissemination of knowledge in hepatology and gastroenterology. This region was the setting for an innovative “educate, test, and treat” community-based HCV program in 73 villages. This acted as a model for the elimination of HCV infection in rural communities and achieved high treatment coverage and cure of the estimated infected adult population [24]. It was also part of the national “100 Million Healthy Lives” program, which aimed to screen the entire adult population in the country between October 2018 and April 2019 [23, 25]. This site was chosen for inclusion in this small pilot study (less than 120 persons) based on this existing collaborative partnership on HCV testing and treatment [24] and not the intended prioritisation of Egypt for the positioning and promotion of HCVST.

### Sample size

As there are no published data on acceptance of HCV self-testing, we made a conservative assumption that 50% of eligible individuals will accept self-testing. To estimate the proportion in this study with a 95% confidence interval based on Wilson’s score method, with +/− 10% margin of error, the minimum sample size of 100 participants will be required.

### Participants

The study participants were recruited consecutively. Patients aged 18 years or older and accompanying adults attending general medical outpatient clinics at the ALPC and Shirbin Hospitals between 5 and 19 May 2019 were invited to participate in this study. Individuals were excluded if they had previous experience with HIV and/or HCV self-testing. All participants who were willing to participate and provided written informed consent were enrolled.

### Test kit

For the purposes of this study, we used the OraQuick® HCV Rapid Antibody Test (OraSure Technologies Inc., Bethlehem, PA, USA), a manually performed, visually read, 20-min immunoassay for the qualitative detection of HCV antibodies in serum, whole blood and oral fluids. Independent evaluation reported 99.4% sensitivity of the OraQuick® HCV Rapid Antibody Test in fingerstick whole blood and 97.6% sensitivity in oral fluids while the specificity was 100% in both specimen types [26]. The test has been prequalified by WHO for professional use [27]. For the study purposes, OraQuick® HCV Rapid Antibody Test was repackaged by the manufacturer for self-testing. It was labeled with the instructions for use, which had been adapted by the manufacturer for self-testing and then translated into Arabic.

### Procedure

Ten hospital staff (one physician, four nurses, three laboratory technicians, and two administrative staff) were trained in the assessment of the HCVST process. Standardized checklists were used to record any errors or difficulties participants experienced when conducting HCVST. A one-week, on-site training session was provided for all study personnel at both sites.

The HCVST process included the following steps. **Pre-testing:** (1) Opening the package, (2) reading the instructions for use, (3) removing the test tube from the test pack, (4) removing the cap from the test tube, (5) placing the tube into the stand, (6) removing the test device from the test pack. **Performing the test:** (7) Correct handling of the device (not touching the flat pad), (8) collecting a sample of oral fluid, (9) placing the test device in the test tube, (10) checking the test device stays in the tube while testing, (11) Monitoring the time while waiting for the result. **Results:** (12) Correctly reading and interpreting the results.

Once a participant had been enrolled, a trained study staff gave a one-to-one, in-person demonstration on how to use the HCVST kit, together with written and pictorial instructions (Additional file 1). Immediately after an in-person demonstration session, participants used the OraQuick® HCV Rapid Antibody Test to perform self-testing in a private room. This was carried out under the observation of a designated staff member experienced in HCV testing. The staff member encouraged the participants to perform the self-testing by themselves, without any assistance, and documented any errors or difficulties observed during the testing procedure, using a product-specific checklist. Assistance was provided by the observers only if the participant had exhausted all attempts to complete the testing step (usually after 15 min of trying without success) and requested help. On completion of testing, the results were first read and interpreted by the participant; immediately afterward the results were independently read and interpreted by a staff member trained in use of the OraQuick® HCV Rapid Antibody Test.

Routine data collected included participants’ demographic characteristics, educational level, and self-reported risk factors for HCV infection. Information on participants’ views and attitudes around HCVST were collected using an interviewer-administered paper-based questionnaire (Additional File 4). The questionnaire topics included the participant’s rating of the ease of use of HCVST, willingness to use HCVST again and recommend it to their family and friends, follow-up actions after receiving a positive/reactive result, and preferred modes of testing in the future. The laboratory technician then conducted a second test using an oral fluid OraQuick® HCV Rapid Antibody Test for professional use.

### Analysis

The usability of HCVST was assessed by calculating the number and percentage of participants with documented errors and also those who experienced difficulties identified by the observer, similar to previous studies of HIVST [28]. Inter-reader concordance for self-test results was calculated as the percentage agreement between the results as interpreted by the participant and the same results as interpreted by the study staff; Cohen’s kappa coefficient was then calculated [29]. Inter-operator concordance was determined as the percentage agreement between the self-testing result reported by the participant and the result of a professional-use test conducted by a trained staff member. Cohen’s kappa coefficient was calculated in two ways: one including invalid results and one excluding invalid results. Acceptability was based on participants’ self-reported views and preferences around HCVST, reported as numbers and percentages.

## Results

### Participant characteristics

**Fig. 1:**
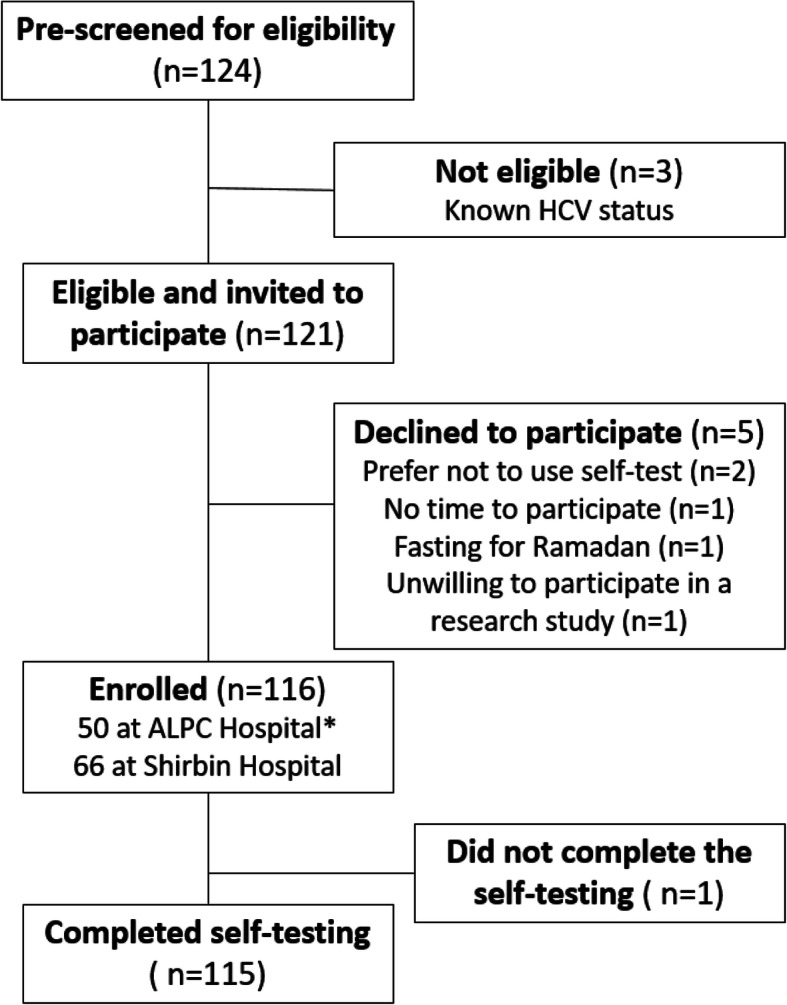
Flowchart of eligibility and enrolment among ALPC* and Shirbin Hospital attendees. *ALPC: Association of Liver Patient Care Hospital

Figure 1 shows the flowchart for study eligibility and enrolment. Between 5 and 19 May 2019 (coinciding with Ramadan and daily fasting), 124 individuals were screened for eligibility; 121 were eligible and invited to participate. Five individuals declined, two of whom indicated an unwillingness to use a self-test as the reason for declining. Table 1 summarizes the demographic characteristics of the 116 enrolled participants: 70 (61%) were male, median age was 39 years (IQR 32–48), 88 (75.8%) had at least secondary school education, while 17 (14.6%) of the participants had received no formal education (7 women and 10 men). Two-thirds of enrolled participants were employed (76, 65.5%) and 91 (78.4%) were married. Twenty-eight (24.1%) participants reported having a household member who was HCV-positive, and the majority of participants (104, 89.6%) were aware that self-testing was available for other medical conditions.

**Table 1:** Baseline demographic characteristics of the 116 participants enrolled in the study

	*n* = 116	%
**Median age, years (IQR)**	39 (32–48)	
**Sex**		
Female	46	39%
Male	70	61%
**Educational level**		
No education	17	14.6%
Primary school	9	7.7%
Secondary school	62	53.4%
College or higher	26	22.4%
Not available	2	1.7%
**Employment status**		
Unemployed	40	34.5%
Employed	76	65.5%
**Marital status**		
Married	91	78.4%
Unmarried	14	12%
Divorced or widowed	10	8.6%
Not available	1	0.8%
**Self-reported exposure (ever) to any of the following risk factors for HCV infection**		
Dental procedure(s)	98	84.5%
Surgical procedure(s)	42	36.2%
Sharing shaving tools or toothbrushes	22	18.9%
Injecting unprescribed drugs or sharing needles	2	1.7%
HCV-positive household member	28	24.1%
None reported	6	5.2%
**Frequency of routine health checks per year**		
More than once per year	32	27.6%
Once per year	19	16.4%
Rarely	49	42.2%
Never	16	13.8%
**Awareness of self-testing**		
Aware that certain kinds of tests can be performed at home	104	89.6%

### Usability of HCV self-testing

Table 2 shows the results of observer assessment of errors, difficulties, and need for assistance during the self-testing process. Only one of the 116 participants was unable to complete the HCVST process and stopped after step 5 (placement of the buffer tube into the stand), due to spillage of the buffer solution. Overall, 102 (88%) participants completed the testing procedure without any errors and 99 (86%) interpreted the results correctly. The most frequently observed errors were incorrect handling of the test device (i.e., a participant touched the flat pad used for specimen collection), not using correct timekeeping, and incorrect interpretation of the test results. In addition, five participants did not utilize the written instructions for use (three were illiterate) (Table 2). Around half of the participants (62, 53.4%) experienced difficulties with at least one step. The most frequently observed difficulties were removing the cap from the test tube (48, 41.4%) and sliding the tube into the stand (21, 18.1%). Less frequent difficulties were opening the package, placing the test device into the tube, and reading the results (Table 2). Assistance was provided to 14 of the 116 participants and four required assistance for more than one step in the testing process.

**Table 2:** Observer assessment of errors (using a product-specific checklist), difficulties, and steps requiring assistance

Observation	% (*n*) of participants
Observed errors at each step using the usability checklist	
**Pre-testing**	
Opening the package	0% (0/116)
Reading/using the instructions for use	4.3% (5/116)
Removing the test tube from the test pack	0% (0/116)
Removing the cap from the test tube	0.8% (1/116)
Placing the tube into the stand	4.3% (5/116)
Removing the test device from the test pack^a^	0% (0/115)
**Conducting the test**	
Touched the flat pad	4.3% (5/115)
Incorrect manipulation to collect oral fluid	0.8% (1/115)
Incorrect placing of the test device in the test tube	0.8% (1/115)
Test device came out of the tube while testing	0% (0/115)
Incorrect timekeeping	5.2% (6/115)
Errors observed during at least one step	12% (14/116)
**Test interpretation**	
Interpreted test results incorrectly (the result read by the study participant was not in agreement with re-reading by a trained staff member)	13.9% (16/115)
**Observed difficulties with testing steps**	
Opening the package	12.1% (14/116)
Removing the cap from the test tube	41.4% (48/116)
Placing the tube into the stand	18.1% (21/116)
Placing the test device into the tube	2.6% (3/115)
Reading and interpreting the results	2.6% (3/115)
Experienced difficulties during at least one step	53.4% (62/116)
**Assistance provided**^b^	
Opening the package	2% (2/116)
Opening and removing the cap from the tube	4% (5/116)
Placing the tube into the stand	7% (8/116)
Placing the test device into the tube^a^	1% (1/115)
Reading the results^a^	2.6% (3/115)
Assistance provided during at least one step	12.1% (14/116)
Completed all testing steps correctly without any assistance and interpreted the test results correctly	72% (84/116)

^a^One participant poured the buffer out of the buffer tube and had to stop the testing procedure, affecting the following observations in this table; ^a^Assistance was provided when requested by a participant after they made multiple efforts to conduct the test unassisted

### Interpretation of self-test results: inter-reader concordance

Overall, 113 of the 115 participants who completed the testing procedure also interpreted their test result, but two were unable to do so. A total of 91 participants read their results as negative, 18 as positive, and 4 as invalid. Three of the 115 self-test results were interpreted as being invalid by the trained study staff (Table 3). One study participant read their invalid result as positive and another read theirs as negative. The results of five tests determined to be positive by the study staff were reported negative by study participants. Two of these five tests produced very faint test lines, and these participants tested negative when subsequently tested by study staff. Four participants reported positive results that were interpreted as negative by trained staff. Overall, inter-reader concordance was 86%, with a Cohen’s kappa value of 0.6.

**Table 3:** Assessment of inter-reader (left panel) and inter-operator (right panel) concordance

	Re-reading by a trained staff member				Re-testing by a trained staff member		
Participant assessment	Positive	Negative	Invalid	Total	Positive	Negative	Invalid
Positive	13	4	1	18	15	3	0
Negative	5	85	1	91	3	88	0
Invalid	2	1	1	4	1	3	0
Unsure	1	1	0	2	1	1	0
Total	21	91	3	115	20	95	0
Concordance (%)	86%				89.5% 92.7%^a^		
Cohen’s kappa	0.6				0.75^a^		
Test failure rate	2.6%						

^a^Excluding invalid results

### Concordance of self-testing and provider-delivered testing results

Participants were re-tested by a study staff member using oral fluid OraQuick® HCV Rapid Antibody Test for professional use and this re-testing yielded 95 negative results and 20 positive results. When all HCVST results reported by participants were compared with results of re-testing by trained study staff, the inter-operator concordance was 89.5%, with a Cohen’s kappa value of 0.67. When invalid results were excluded from the analysis, the concordance was 92.7%, with a Cohen’s kappa value of 0.75 (Table 3).

### Acceptability and user attitudes toward HCV self-testing

**Fig. 2:**
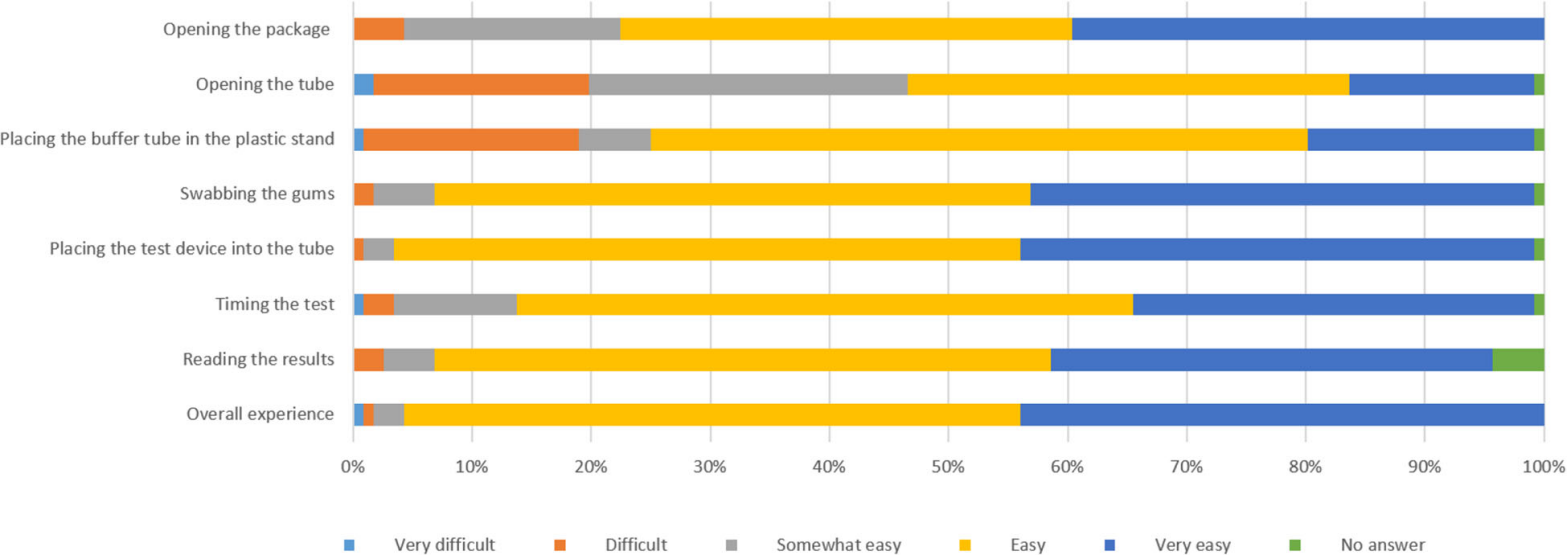
Participants’ perceptions of HCV self-test usability at different steps

Prior to conducting the HCVST procedure, more than 95% of participants expressed a willingness to use an HCV self-test if this option was available. After completing HCVST, two participants (1.7%) rated the overall experience as “difficult” or “very difficult”; both of these individuals lacked a formal education and were also unable to tell the time without assistance. About 18% of participants found both opening the buffer tubes and placing the tube in the plastic stand to be difficult (Fig. 2).

Table 4 summarizes the findings of the interviewer-administered assessment of participant post-testing feedback. The majority of participants (112/116, 96.5%) reported that they would use an HCV self-test again if it were available. Of the four who indicated they would not use an HCVST again, reasons included the complexity of the procedure (one participant) and having to pay for the test themselves (two participants).

**Table 4:** Participant views and preferences regarding HCVST

**Pre-test acceptability (before self-testing)**	
Number (%) of eligible individuals who agreed to participate and perform HCVST	116/121 (95.8%)
Number (%) of participants who would use HCVST if it were available	111/116 (95.6%)
**Post-testing acceptability (after self-testing)**	**Participants,** ***n*** **(%)** ***n*** **= 116**
Would use the HCVST again	
Yes	112 (96.5%)
No	4 (3.4%)
Would recommend the HCVST to family members/friends	
Yes	115 (99.1%)
No	1 (0.9%)
Would take the test to family members/friends	
Yes	107 (92.2%)
No	1 (0.9%)
Not sure	8 (6.9%)
**Preferences with regard to HCVST**	**Participants,** ***n*** **(%)** ***n*** **= 116**
Preferred approach to test for HCV in the future	
By myself at home	78 (67.2%)
By myself at a health center	10 (8.6%)
In a community center by a healthcare worker	27 (23.3%)
In a screening campaign	1 (0.8%)
Preferred sample type	
Prefer oral fluid-based test	75 (64.6%)
Prefer blood-based test	28 (24.1%)
No preference	13 (11.2%)
Steps they would take if the results of a self-test were positive	
Contact a healthcare facility	113 (97.4%)
Contact a pharmacy	1 (0.9%)
Perform a confirmatory test	1 (0.9%)
Seek advice from a family member/community	1 (0.9%)
Do not know	0 (0%)
**Knowledge about HCV treatment**	
Know that HCV can be cured	78 (67.2%)
Know that there is a treatment but not sure about the cure	27 (23.3%)
Not sure if there is treatment	10 (8.69%)
There is no treatment or cure	1 (0.9%)

Nearly all participants (115/116, 99%) said they would recommend the test to their family and friends, two-thirds (78, 67.2%) reported that testing at home would be their preferred method of testing for HCV in the future, while 27 (23.3%) expressed a preference for testing by a healthcare provider. The most common reasons for a preference for HCVST were protection of privacy (35, 30.2%) and the flexibility to conduct a test at any time (57, 49.1%), while the main disadvantage noted was the absence of counselling (Additional File 3). Six participants (5%) also indicated that they were not confident in the HCVST results (Additional File 3). There was a preference for oral fluid-based HCVST among 75 (64.6%) participants and blood-based testing among 28 (24.1%). The majority of participants were aware that they would need to contact a health facility in the event of a positive HCV self-test result (113, 97%), and 78 participants (67%) were aware that HCV infection is curable.

## Discussion

This is one of the first studies globally to report on the usability and acceptability of an oral fluid-based HCV antibody self-test among the general population in an LMIC setting. The 116 study participants were enrolled from attendees at two outpatient clinics in the Nile Delta region of Egypt, a region with a high HCV prevalence but also a high level of awareness of HCV infection. Overall, our study showed high usability and acceptability of HCVST. The majority of participants were able to correctly perform HCVST, following a short one-to-one demonstration on how to use the test. Although most participants (88%) conducted the HCVST process without any mistakes and interpreted the results correctly, more than half (53.4%) were observed to have difficulties with at least one step, and some participants (12%) requested assistance (four required assistance with more than one step in the testing process). The most common errors were incorrect handling of the test device (i.e., a participant touched the flat pad), incorrect timekeeping, and misinterpretation of test results. The most frequently observed difficulties related to removing the cap from the test tube and sliding the tube into the stand.

Our findings are broadly consistent with those from earlier, comparable studies of HIVST [30–33]. In a 2014 study of the usability of five different HIV self-test devices in unsupervised settings in Kenya, Malawi, and South Africa, 15% of participants made more than one error with an oral fluid self-test [34]. Similar user errors and difficulties have been reported in other HIVST studies [12, 13, 28], especially with early prototype test kits and instructions for use that were not yet optimized for self-testing [30]. The most common errors with oral fluid HIVST kits were incorrect swabbing of the gums and misinterpretation of the results, particularly those with faint positive lines. With blood-based HIVST kits, difficulties in sample collection were documented in 5 to 31% of participants, especially among those from high-risk populations [12, 31]. Generally, fewer user errors were reported when there was in-person observation, video recording of participants, provision of additional training, or direct supervision [12].

In the present study, overall inter-reader agreement was 86%, with a Cohen’s kappa value of 0.6. Three participants yielded invalid self-test results, although they had all collected their sample correctly and read the results after waiting for the appropriate length of time (Additional File 2). Five participants reported positive test results as negative and four reported negative results as positive. More than half of these misinterpretations (5 out of 9) were among participants with low levels of education or literacy (Additional File 2). The two participants who were unable to interpret their test results were both aged more than 60 years and had only received primary school education. The inter-operator concordance (i.e., comparing self-test results with the results of a rapid test performed by a provider) was 92.7%, with a Cohen’s kappa value of 0.75. These values fall within the range of 85.4 to 100% and 0.28 to 0.99, respectively, reported in a previous systematic review of HIVST studies [13]. The pooled kappa value in this systematic review also showed comparable results for directly assisted (0.98, 95% CI 0.96–0.99) and unassisted HIVST (0.97, 0.96–0.98), suggesting that self-testers can perform HIVST as well as trained providers. In an HIVST study with relatively low levels of agreement (kappa value 0.47, − 0.04 to 0.97), conducted in rural Zimbabwe, the study investigators attributed the poor performance to both low levels of literacy in the population tested and suboptimal instructions for use [13]. While in our study the overall concordance rate was high, we found three false-negative and three false-positive results, indicating that additional support for self-testers may be needed in the initial phases of implementation.

There was a high level of pre- and post-test acceptability of HCVST in our study, consistent with reports for HIVST [10, 12]. The majority of study participants rated the HCVST procedure as easy or very easy and stated that they would be willing to use a self-test again and recommend it to their friends and family. The most common reasons expressed for preferring to use a self-test were greater privacy and the possibility to perform a self-test at any time. The majority of participants were also aware of the need to contact health services for confirmatory viral load testing and to determine their eligibility for treatment. Although we used an oral fluid-based test, 24% of participants expressed a preference for blood-based assays. While the reasons for this preference were not sought in our study, extensive research into HIVST has shown that people express no clear preference for blood versus oral fluid HIVST kits. Some people express a preference for oral fluid tests because they are pain-free and easy to perform, while others prefer blood-based tests because of their perceived greater accuracy [12, 35–37]. Recent WHO guidance on HIVST encourages country programs to offer a choice in the type of self-test kits offered and sample types collected, promote supplier diversity, and address the preferences of different population groups [11]. Further work is ongoing to assess the usability of blood-based self-tests for HCV.

This study has several limitations that must be considered when interpreting the findings. The sample size of 116 participants was small, and the study was based on the use of an oral fluid test only. The findings may therefore not be generalizable to the larger HCV-infected population in the community in Egypt, or to other sample types. About 75% of the participants enrolled in this study had at least completed secondary school, however, education and literacy levels in other rural populations in Egypt may be lower. The rates of errors and difficulties with self-testing procedure could be higher in populations with lower educational levels. The provision of an initial in-person demonstration for all participants in this study, combined with the observation of participants during the HCVST process and availability of assistance, may also have influenced how the HCVST procedure was conducted, resulting in fewer errors and difficulties. Egypt has a well-established, effective, and free national HCV testing, care, and treatment program [3]. High levels of awareness about this disease and ready access to confirmatory testing and treatment in Egypt is likely to have contributed to higher levels of acceptability than in settings and populations without such a program. For example, a recent study among PWID in the UK found a lower acceptability of HCVST; perceived barriers in access to confirmatory testing and treatment, as well as a lack of post-testing counselling and the need to cope with test results in isolation, were among the key concerns expressed [17].

What are the implications of our findings for future HCVST implementation projects in other countries and settings? First, there is a need to minimize errors and difficulties related to self-testing, by simplifying test procedures, improving test devices, optimizing instructions for use, and providing support tools. This may include the use of instructional videos as well as virtual and even in-person assistance for some individuals or populations, for example those with low literacy levels. Additional support tools to accompany further roll-out of HCVST and linkage to care may include telephone hotlines, interactive resources in social media, and mobile apps. Such tools have been developed and successfully implemented during the roll-out of HIVST [8, 11].

Self-testing provides a convenient alternative to provider-delivered testing, however, its higher cost, lack of face-to-face counselling and poor linkage and access to further care may be important barriers to HCVST implementation. Although randomized clinical trials have shown that HIVST can achieve linkage rates comparable with standard testing following a reactive result [11], HCV diagnosis requires a two-step process, with viral load confirmation following positive serology test result, and HCVST will require specific strategies and messaging to promote linkage to care.

In addition to the four other recently completed HCVST studies that used the same protocol as this study, in high-risk populations in Vietnam, China, Georgia, and Kenya, there is a need to evaluate a range of oral fluid- and blood-based HCVST assays in different populations and settings. Additional studies are needed to compare the HCVST approach with other community- and facility-based HCV testing to identify the optimal positioning of self-testing for promoting access to testing and treatment.

Overall, our small pilot study demonstrated the feasibility of assisted self-testing for HCV in a general population sample from a semi-rural setting in the high HCV prevalence Nile Delta region. Importantly, this study does not inform the wider acceptability and applicability of HCVST to the generalised HCV epidemic in Egypt. There has already been a substantial investment in HCV case-finding as part of the national HCV programme in Egypt, with more than 60 million people tested through the recent national campaign, demonstrating the feasibility and success of a comprehensive population-wide screening approach to achieve disease elimination. However, HCVST may still have a potential role to play in promoting access to testing among those not yet reached. This could include young people, college students, and certain marginalized populations, such as MSM and PWID, who may not wish to test through existing testing services, or those with limited geographic access to healthcare facilities.

## Supplementary Information

**Additional file 1: Supplementary Fig. 1.** Manufacturer’s instructions for use in Arabic and in pictures. Images showing instructions for use, in Arabic and in pictures.

**Additional file 2: Supplementary text: Data collection forms.**. Screening log. Baseline questionnaire. Checklist for the self-testing process. Post-test questionnaire

**Additional file 3: Supplementary Table 1.** Additional perceptions on HCV self-testing. Additional responses of participants about their opinions of HCV self-testing

**Additional file 4: Supplementary Table 2.** Discordances in results reported by participants and results obtained by re-reading (interpreted by the trained staff) and re-testing (with professional use kit, by the trained staff). Table showing discordances in results reported by participants and results obtained by re-reading and re-testing by trained staff.

## Data Availability

The datasets used and/or analysed during the current study are available from the corresponding author on reasonable request.

## Abbreviations

ALPC: Association of liver patient care
DAA: Direct-acting antiviral
FIND: Foundation for innovative new diagnostics
HCVST: Hepatitis C virus self-test
HIV: Human immunodeficiency virus
HIVST: HIV self-test
LMIC: Low- and middle-income country
MSM: Men who have sex with men
PWID: People who inject drugs
RDT: Rapid diagnostic test
WHO: World health organization
