# Supplementary material for: Usability and acceptability of self-testing for hepatitis C virus infection among the general population in the Nile Delta region of Egypt
Source: BMC Public Health. 2021 Jun 22;21:1188. doi: 10.1186/s12889-021-11169-x (PMC8218412; doi:10.1186/s12889-021-11169-x)
Supplement: Supplementary file 1 — Additional file 1: Supplementary Fig. 1. Manufacturer’s instructions for use in Arabic and in pictures. Images showing instructions for use, in Arabic and in pictures. [file 12889_2021_11169_MOESM1_ESM.docx]

**Supplementary figure 1.** Manufacturer’s Instructions for Use in Arabic
