# Supplementary material for: Usability and acceptability of self-testing for hepatitis C virus infection among the general population in the Nile Delta region of Egypt
Source: BMC Public Health. 2021 Jun 22;21:1188. doi: 10.1186/s12889-021-11169-x (PMC8218412; doi:10.1186/s12889-021-11169-x)
Supplement: Supplementary file 2 — Additional file 2: Supplementary text: Data collection forms.. Screening log. Baseline questionnaire. Checklist for the self-testing process. Post-test questionnaire [file 12889_2021_11169_MOESM2_ESM.docx]

**Supplementary Text.** Data collection forms

**Screening log**

| \| **Nr.** \| **Screening date**  **(DD-MMM-YY)** \| **Sex** \| **Age (years)** \| **Unknown HCV serology status?** \| **Agreed?** \| **Reason if not agreed** \| \| --- \| --- \| --- \| --- \| --- \| --- \| --- \| \|  \| -- \| M F \|  \| YES  NO \| YES  NO \|  Do not have time to participate in the study   Prefer to test elsewhere  Prefer not to use self-test  Prefer not to say Does not meet eligibility criteria   Fasting Other: ______________________________________ \| \|  \| -- \| M F \|  \| YES  NO \| YES  NO \|  Do not have time to participate in the study   Prefer to test elsewhere  Prefer not to use self-test  Prefer not to say Does not meet eligibility criteria   Fasting Other: ______________________________________ \| \|  \| -- \| M F \|  \| YES  NO \| YES  NO \|  Do not have time to participate in the study   Prefer to test elsewhere  Prefer not to use self-test  Prefer not to say Does not meet eligibility criteria   Fasting Other: ______________________________________ \| \|  \| -- \| M F \|  \| YES  NO \| YES  NO \|  Do not have time to participate in the study   Prefer to test elsewhere  Prefer not to use self-test  Prefer not to say Does not meet eligibility criteria   Fasting Other: ______________________________________ \| |
| --- | --- | --- | --- | --- | --- | --- | --- | --- | --- | --- | --- | --- | --- | --- | --- | --- | --- | --- | --- | --- | --- | --- | --- | --- | --- | --- | --- | --- | --- | --- | --- | --- | --- | --- | --- |

**Baseline questionnaire**

| 1. **Demographic data of the participant** | | | | |
| --- | --- | --- | --- | --- |
| **Site of the study/enrolment**  🞎 Shirbin 🞎 APLC | **Date of enrolment:**  *(dd/mm/yy)*  **…………./…………./…………..** | | **Full name of the Observer/Interviewer:** | |
| **Gender:**  🞎 Female 🞎 Male | **Age:**  **___________**years | | **Country/Governorate/Village of Provenance:** | |
| **Marital status:**  🞎 Married or living with a partner  🞎 widow  🞎 divorced/separated (not living together)  🞎Never married | **Educational Background:**  *Indicate the highest educational level received*  🞎 Illiterate 🞎 Primary school (6 years) 🞎 Intermediate school (6 + 3 years) Secondary school (6 + 3 + 3 years) 🞎 College | | | |
| **How many people live in the household?**  Total**:___**  Adults**:____**  Children**:____** | **Main reason for your visit today?** 🞎 Screening for hepatitis 🞎 Accompanying another person 🞎 Regular health check 🞎Other reason: ________________________________🞎 Doesn’t answer | | | **Main Occupation:** |
| 1. **HEPATITIS C TESTING AND MORBIDITIES** | | | | |
| **How often do you come to health facilities to check your health?** 🞎 More than 1 time per year  🞎 1 time per year  🞎 **Rarely (once in 2 or more years)**  🞎 Never | **Please select all that apply. Have you have received or engaged in**  🞎 Injecting unprescribed drugs  🞎Sharing needles  🞎 A surgical procedure 🞎 a dental procedure 🞎 Sharing shaving tools or toothbrushes  🞎 Make a tattoo | **Did somebody in your household tested for HCV?**  🞎 No, never.  🞎 Yes, more than 1 year ago  🞎 Yes, in the past 12 months  **If yes, what was the results of the testing?**  🞎 Positive 🞎 Negative 🞎 Don’t know | | |
| **Do you know that there are some tests that you can do at home to check for medical conditions (e.g. pregnancy test, glucose test)?** 🞎 No 🞎 Yes  **If such a test was available to check for hepatitis C, would you be willing to use it?** 🞎 No 🞎 Yes | | | | |
| **Additional Observations/Comments:** | | | | |

**CHECKLIST ON SELF-TESTING PROCESS**

| **Review during THE proceedure** |
| --- |
| **Name of the reviewer:** |
| \| 1. **CHECKLIST** \| \| \| --- \| --- \| \| 1. Did the study participant open the pouch and take all of the contents out? \| 🞏 Yes 🞏No  If no, why: _________________________ \| \| 1. Did the study participant read/use the instructions for use? \| 🞏 Yes 🞏No  If no, why: _________________________ \| \| 1. Did the study participant to remove the test tube from the test pack? \| 🞏 Yes 🞏No  If no, why: _________________________ \| \| 1. Did the study participant remove the cap from the test tube? \| 🞏 Yes 🞏No  If no, why: _________________________ \| \| 1. Did the study participant place the test tube in the test stand? \| 🞏 Yes 🞏No  If no, why: _________________________ \| \| 1. Was the study participant able to remove the test device from the test pack? \| 🞏 Yes 🞏No  If no, why: _________________________ \| \| 1. Did the study participant touch the flat pad? \| 🞏 Yes 🞏No  If yes, why: _________________________ \| \| 1. Did the study participant collect the sample correctly (1x upper and 1x lower swab of gums)? \| 🞏 Yes 🞏No  If no, why: _________________________ \| \| 1. Did the study participant place the test device in the test tube correctly? \| 🞏 Yes 🞏No  If no, why: _________________________ \| \| 1. Did the study participant use a time keeping device (Clock, watch, timer)? \| 🞏 Yes 🞏No  If no, why: _________________________ \| \| 1. Did the study participant read the device results between 20 and 40 minutes after placing the device in the test tube? \| 🞏 Yes 🞏No  If no, why: _________________________ \| |
| 1. **ADDITIONAL DESCRIPTION DURING THE TEST** |
| **1.**  **Mark ALL the problems or mistakes observed when performing the test** |
| 🞎 Rubbed the wrong part of the mouth  🞎 Spilt the fluid from the tube  🞎 Sliding the tube into the stand  🞎 Collecting wrongly the oral fluid  🞎 Touched the flat pad  🞎 Test device came out of the tube while testing  🞎 Reading the results before time  🞎 Not able to identify the test line or the control line  🞎 Mistook the control line for the test line  🞎 No problems or mistakes observed  🞎 Any other  Specify:___________________________________________________________________________________ |
| **2. Which part of self-testing you observed was the most difficult to perform? (Mark one or two)** |
| 🞎 Opening the package  🞎 Opening the tube  🞎 Sliding the tube into the stand  🞎 Placing the test device into the tube  🞎 Reading the results  🞎 All the steps were easy  🞎 All the steps were difficult |
| **3. Was the testing procedure completed?** |
| 🞎 Yes 🞎 No |
| **4. Was assistance provided? If yes, at which step:** |
| 🞎 No  🞎 Yes, opening the package  🞎 Yes, opening the tube  🞎 Yes, sliding the tube into the stand  🞎 Yes, placing the test device into the tube  🞎 Yes, reading the results |
|  |
| 1. **AFTER THE TEST** |
| **1a. To the participant: How easy or difficult was it to understand the written instructions?** |
| 🞎 Very easy 🞎 Somewhat easy 🞎 Somewhat difficult 🞎 Very difficult 🞎 Not used |
| **1b. To the participant: How easy or difficult was it to understand the pictorial instructions?** |
| 🞎 Very easy 🞎 Somewhat easy 🞎 Somewhat difficult 🞎 Very difficult 🞎 Not used |
| **2. To the participant: How useful were written or pictorial instruction during the testing procedure?** |
| 🞎 Very much 🞎 Somewhat 🞎 A little 🞎 Not at all |
| **3. To the participant: Thinking about the test, how did you find performing each of these steps:** |
| \|  \| **Very difficult** \| **Difficult** \| **Slightly difficult** \| **Slightly easy** \| **Easy** \| **Very easy** \| \| --- \| --- \| --- \| --- \| --- \| --- \| --- \| \| **Opening the package** \| ⃝ \| ⃝ \| ⃝ \| ⃝ \| ⃝ \| ⃝ \| \| **Opening the tube** \| ⃝ \| ⃝ \| ⃝ \| ⃝ \| ⃝ \| ⃝ \| \| **Sliding the tube into the stand** \| ⃝ \| ⃝ \| ⃝ \| ⃝ \| ⃝ \| ⃝ \| \| **Swabbing the gums** \| ⃝ \| ⃝ \| ⃝ \| ⃝ \| ⃝ \| ⃝ \| \| **Placing the device into the tube** \| ⃝ \| ⃝ \| ⃝ \| ⃝ \| ⃝ \| ⃝ \| \| **Timing the test** \| ⃝ \| ⃝ \| ⃝ \| ⃝ \| ⃝ \| ⃝ \| \| **Reading the results** \| ⃝ \| ⃝ \| ⃝ \| ⃝ \| ⃝ \| ⃝ \| |
| **5. To the participant: Please rate your overall experience with self-test** |
| 🞎 Very easy 🞎 Somewhat easy 🞎 Neutral 🞎 Somewhat difficult 🞎 Very difficult |

**POST-TESTING QUESTIONNAIRE**

*Thank you for agreeing to participate today and give your informed consent. I would like to ask you about your experiences of oral self-assessment for HCV self-testing. All your answers will remain confidential and you do not have to answer to questions that you do not want. There are no rights or wrong answers to these questions. Please free to ask questions anytime during the interview and we can stop at any time. Thank you again for your participation.*

| **Acceptability and feasibility of HCV self-testing in general population in Egypt** |
| --- |
| **Name of the reviewer:** |
| 1. **In your opinion, is HCV ST an accurate test? (Do you trust these results?)** |
| 🞎 Very accurate 🞎 Somewhat accurate 🞎 Neutral 🞎 Do not know |

| 1. **What made you decide to have an HCV test today? (Read and Circle all that apply)** | | | | |
| --- | --- | --- | --- | --- |
| **Statement** | **Yes** | **No** | **DK** | **NR** |
| a. *I got the information at my community/village* | 1 | 0 | 88 | 99 |
| b. *A doctor/nurse recommended it* | 1 | 0 | 88 | 99 |
| c. *I knew that treatment was available* | 1 | 0 | 88 | 99 |
| d. *I was sick* | 1 | 0 | 88 | 99 |
| e. *A partner, family or friends advised to have a test* | 1 | 0 | 88 | 99 |
| f. *TV or radio messages* | 1 | 0 | 88 | 99 |
| g. *It was necessary for a job* | 1 | 0 | 88 | 99 |
| h. *It was necessary* *to donate blood* | 1 | 0 | 88 | 99 |
| i. *It was required for administrative (ex. employment) purposes* | 1 | 0 | 88 | 99 |
| j. *You were worried about a sexual contact* | 1 | 0 | 88 | 99 |
| k. You were worried about a potential risk you consider for infection | 1 | 0 | 88 | 99 |
| l. *You heard that you can take the test and get the results in the same day* | 1 | 0 | 88 | 99 |
| m. It was necessary to apply for health insurance or life insurance | 1 | 0 | 88 | 99 |
| n. You were encouraged and supported by someone who had been tested | 1 | 0 | 88 | 99 |
| o. Chiefs, religious leaders or political leaders got tested and this convinced you | 1 | 0 | 88 | 99 |
| p. *Other (please specify)*  *________________________________________________________________* | 1 | 0 | 88 | 99 |

| 1. **Overall, how satisfied were you with the HCV self-testing process?** |
| --- |
| 🞎 Very satisfied 🞎 Somewhat satisfied 🞎 A little satisfied 🞎 Not satisfied at all |
| 1. **Would you recommend HCV self-testing to a friend or family member?** |
| 🞎 Yes 🞎 No  **Why?**  __________________________________________________________________________________________ |
| 1. **How would you feel about taking tests home for your friends or family?** |
| 🞎 Positive 🞎 Prefer not 🞎 Not sure  **Why?**  __________________________________________________________________________________________ |
| 1. **Would you like to use this hepatitis C self-test again?** |
| 🞎 Yes 🞎 No  **___________________________________________________________________________________________** |
| 1. **In your opinion, what are the advantages of self-testing for hepatitis C?** |
| 🞎 The test can be performed in privacy 🞎 No need to come to a clinic 🞎 I can test myself anytime 🞎 Not sure  🞎 Other, specify: |
| 1. **Which are the disadvantages of self-testing for hepatitis C?** |
| 🞎 Difficult to perform 🞎 No confidence in test results 🞎 Getting the results alone (no counselling) 🞎 Need to pay for the test 🞎 Not sure  🞎 Other, specify:  ___________________________________________________________________________________________ |
| 1. **What would be your preferred mode to test for Hepatitis C in the future? *Read the options*** |
| 🞎 By myself at home 🞎 By myself at a Health Center 🞎 In a Community center by Health Care Worker  🞎 In a screening campaign 🞎 Taking a regular sample at a HF 🞎 Any, No specific preference |
| **12. Would you prefer a test collecting a blood sample from a fingerstick or no special preference?** |
| 🞎 Yes 🞎 No 🞎 Indifferent |
| 1. **In the case of doing the test by yourself, would you be comfortable on reading any result alone?** |
| 🞎 Yes 🞎 No  **Why?**  __________________________________________________________________________________________ |
| 1. **What would you do if your HCV self-test is giving a positive result (indicating a likelihood of having hepatitis C infection)?** |
| 🞎 Contact health facility 🞎 Contact pharmacy 🞎 Do a confirmatory test 🞎 Seek advice from family members/community 🞎 Seek advice from a religious representative (e.g. priest) 🞎 Do not know 🞎 Other: please specify: __________________________________________ |
| 1. **Do you know if people can be treated and cured for Hepatitis C?** |
| 🞎 Yes, there is a treatment, but not sure about cure 🞎 Yes, there is treatment and cure 🞎 Not sure if treatment or cure 🞎 There is no treatment or cure 🞎 No idea |
| 1. **Do you know if there is treatment available for Hepatitis C in your village/near your village?** |
| 🞎 Yes 🞎 Yes, but not nearby 🞎 No 🞎 No idea |
| 1. **Interviewer comments on specific questions, respondent, interview, any additional** |
|  |
