# Supplementary material for: Usability and acceptability of self-testing for hepatitis C virus infection among the general population in the Nile Delta region of Egypt
Source: BMC Public Health. 2021 Jun 22;21:1188. doi: 10.1186/s12889-021-11169-x (PMC8218412; doi:10.1186/s12889-021-11169-x)
Supplement: Supplementary file 3 — Additional file 3: Supplementary Table 1. Additional perceptions on HCV self-testing. Additional responses of participants about their opinions of HCV self-testing [file 12889_2021_11169_MOESM3_ESM.docx]

**Supplementary Table 1.** Additional Perceptions on HCV Self-Testing

|  | Female  n (%) | Male  n (%) | Total  n (%) |
| --- | --- | --- | --- |
| Overall, how satisfied were you with the HCV self-testing process?  (n=116) | | | |
| Very satisfied | 20 (43.4) | 35 (50) | 55 (47.4) |
| Somewhat satisfied | 22 (47.8) | 33 (47.1) | 55 (47.4) |
| A little satisfied | 3 (6.5) | 2 (2.8) | 5 (4.3) |
| Not satisfied at all | 1 (2.1) | 0 | 1 (0.8) |
| In your opinion, what are the advantages of self-testing for hepatitis C?  (more than one answer by participant possible) | | | |
| The test can be performed in privacy | 13 (27.7) | 23 (28) | 36 (27.9) |
| No need to come to a clinic | 8 (17) | 20 (24.4) | 28 (21.7) |
| I can test myself anytime | 25 (53.2) | 34 (41.5) | 59 (45.7) |
| Not sure/None | 1 (2.1) | 4 (4.8) | 5 (3.9) |
| Other | 0 | 1 (1.2) | 1 (0.7) |
| Which are the disadvantages of self-testing for hepatitis C?  (more than one answer by participant possible) | | | |
| Difficult to perform | 1 (2.1) | 1 (1.4) | 2 (1.7) |
| No confidence in test results | 3 (6.4) | 3 (4.3) | 6 (5.1) |
| Getting the results alone (no counselling) | 18 (38.3) | 30 (42.8) | 48 (41) |
| Need to pay for the test | 8 (17) | 10 (14.3) | 18 (15.4) |
| Not sure | 17 (36.2) | 24 (34.3) | 41 (35) |
| Other | 0 | 2 (2.8) | 2 (1.7) |
| In the case of doing the test by yourself, would you be comfortable on reading any result alone?  (n=116) | | | |
| Yes | 43 (93.5) | 68 (97.1) | 111 (95.7) |
| No | 3 (6.5) | 2 (2.9) | 5 (4.3) |
| Do you know if there is treatment available for Hepatitis C in your village/near your village?  (n=116) | | | |
| Yes, nearby | 24 (52.2) | 38 (54.3) | 62 (53.4) |
| Yes, but not nearby | 22 (47.8) | 30 (42.8) | 52 (44.8) |
| No | 0 | 2 (2.8) | 2 (1.7) |
|  |  |  |  |
