# Supplementary material for: Usability and acceptability of self-testing for hepatitis C virus infection among the general population in the Nile Delta region of Egypt
Source: BMC Public Health. 2021 Jun 22;21:1188. doi: 10.1186/s12889-021-11169-x (PMC8218412; doi:10.1186/s12889-021-11169-x)
Supplement: Supplementary file 4 — Additional file 4: Supplementary Table 2. Discordances in results reported by participants and results obtained by re-reading (interpreted by the trained staff) and re-testing (with professional use kit, by the trained staff). Table showing discordances in results reported by participants and results obtained by re-reading and re-testing by trained staff. [file 12889_2021_11169_MOESM4_ESM.docx]

**Supplementary Table 2.** Discordances in results reported by participants and results obtained by re-reading (interpreted by the trained staff) and re-testing (with professional use kit, by the trained staff)

| **Study ID** | **Manipulation to**  **collect oral fluid** | **Assistance to read the result** | **Participant’s result reading** | **Re-reading** | **Re-testing** | **Observations for discordant results** |
| --- | --- | --- | --- | --- | --- | --- |
| HC012010007 | Correct | No | Negative | Invalid | Negative | Difficulties manipulating the tube were observed, no additional issues reported |
| HC012010014 | Correct | Yes | Positive | Negative | Negative | Wrong interpretation of the result by the participant |
| HC012010015 | Correct | No | Invalid | Invalid | Negative | Participant forgot to read the instructions; the second test provided a more adequate result |
| HC012010027 | Correct | Yes | Unsure | Positive | Positive | Participant did not note the time for the procedure (no clock available); (results were read at the correct time by the observer) |
| HC012010044 | Correct | No | Positive | Negative | Negative | Wrong interpretation of the result by the participant |
| HC012010045 | Correct | No | Negative | Positive | Positive | Wrong interpretation of the result by the participant |
| HC012010052 | Correct | Yes | Unsure | Negative | Negative | Participant unable to read the result (60 years old, finished primary school) |
| HC012010053 | Correct | No | Invalid | Invalid | Negative | Correct manipulation and correct result interpretation; the second test provided a more adequate result |
| HC012010066 | Correct | No | Other | Negative | Negative | Participant unable to read the result. Reported all the steps as easy. |
| HC012010105 | Correct | No | Positive | Invalid | Negative | Participant did not read the instructions; did not use the stand, and did not hold the tube for the time required (participant was illiterate) Additionally, the participant mentioned they were not satisfied at all with the test |
| HC012010108 | Correct | No | Negative | Positive | Negative | Participant was illiterate. There was also the presence of a faint line in the first test, considered by the observer to be weak positivity |
| HC012010109 | Correct | No | Negative | Positive | Positive | All steps were reported as easy by the participant. Presence of a faint line, probably not distinguished by the participant |
| HC012010110 | Correct | No | Positive | Negative | Positive | There was the presence of a faint line (not considered by the observer). The participant was illiterate. |
| HC012010117 | Correct | No | Negative | Positive | Negative | The participant struggled during the procedure and touched the flat pad. Presence of a faint line in the first test was considered by the observer to be weak positivity |
| HC012010121 | Correct | No | Invalid | Positive | Negative | There was the presence of a faint line in the first test, considered by the observer to be weak positivity. The participant was illiterate |
| HC012010127 | Correct | No | Invalid | Positive | Positive | Wrong interpretation of the result by the participant |
| HC012010130 | Correct | No | Negative | Positive | Positive | There was a faint line, probably not distinguished by the participant |
| HC012010145 | Correct | No | Positive | Negative | Negative | Wrong interpretation of the result by the participant. The participant was illiterate. |
